# Heme metabolism in nonerythroid cells

**DOI:** 10.1016/j.jbc.2024.107132

**Published:** 2024-03-02

**Authors:** Luke S. Dunaway, Skylar A. Loeb, Sara Petrillo, Emanuela Tolosano, Brant E. Isakson

**Affiliations:** 1 Robert M. Berne Cardiovascular Research Center, University of Virginia School of Medicine, Charlottesville, Virginia, USA; 2 Department of Molecular Physiology and Biophysics, University of Virginia School of Medicine, Charlottesville, Virginia, USA; 3 Deptartment Molecular Biotechnology and Health Sciences and Molecular Biotechnology Center “Guido Tarone”, University of Torino, Torino, Italy

**Keywords:** heme, heme oxygenase, oxidation-reduction (redox), iron, metalloprotein

## Abstract

Heme is an iron-containing prosthetic group necessary for the function of several proteins termed “hemoproteins.” Erythrocytes contain most of the body’s heme in the form of hemoglobin and contain high concentrations of free heme. In nonerythroid cells, where cytosolic heme concentrations are 2 to 3 orders of magnitude lower, heme plays an essential and often overlooked role in a variety of cellular processes. Indeed, hemoproteins are found in almost every subcellular compartment and are integral in cellular operations such as oxidative phosphorylation, amino acid metabolism, xenobiotic metabolism, and transcriptional regulation. Growing evidence reveals the participation of heme in dynamic processes such as circadian rhythms, NO signaling, and the modulation of enzyme activity. This dynamic view of heme biology uncovers exciting possibilities as to how hemoproteins may participate in a range of physiologic systems. Here, we discuss how heme is regulated at the level of its synthesis, availability, redox state, transport, and degradation and highlight the implications for cellular function and whole organism physiology.

Heme is an iron-containing prosthetic group necessary for the function of several proteins termed “hemoproteins.” Eukaryotes have four heme types *a*, *b*, *c*, and *o* with heme *b* (iron protoporphyrin IX [PPIX], protoheme) being the most prevalent form (1). With few exceptions, this review will discuss heme *b*. Erythrocytes contain the majority of the body’s heme in the form of hemoglobin and also have high concentrations of free heme (∼20 μM) (2), while nonerythroid cells have an estimated ∼25 to 300 nM of cytosolic labile heme (3). The highest levels of nonerythroid heme are found in the liver, which is necessary to maintain high expression of cytochromes P450 (4). However, hemoproteins are found in almost every subcellular compartment (3) and are integral in essential cellular processes such as oxidative phosphorylation (5), amino acid metabolism (5, 6), and transcriptional regulation (7, 8). As such, the (dys)regulation of heme has a major impact on each of these systems. Here, we review the pathways by which heme is synthesized, transported, and metabolized and highlight the functional relevance of these pathways in nonerythroid cells.

## Heme synthesis

All cells must facilitate some degree of heme synthesis to account for essential hemoproteins, such as cytochromes in the electron transport chain. However, given the variation in intracellular hemoprotein requirements, not all cell types synthesize heme to the same degree. Erythrocytes produce around 85% of the organismal heme content to allow for complete hemoglobinization (4). The majority of the remaining percentage of heme synthesis is facilitated within the liver (specifically, in hepatocytes), which is highly enriched in cytochromes P450 (4). These proteins are one of the largest hemoprotein families and are essential for xenobiotic detoxification and steroid metabolism (9). Of note, many of the tissues with high expression of δ-aminolevulinic acid synthase (ALAS), the rate-limiting enzyme in heme synthesis, also have the highest expression of cytochromes P450 (10). Specifically, ALAS was found to be elevated in the testis, endocrine glands, exocrine glands, and respiratory tract, suggesting that these tissues are other major sites of heme synthesis (10).

### Heme biosynthetic pathway

**Figure 1:**
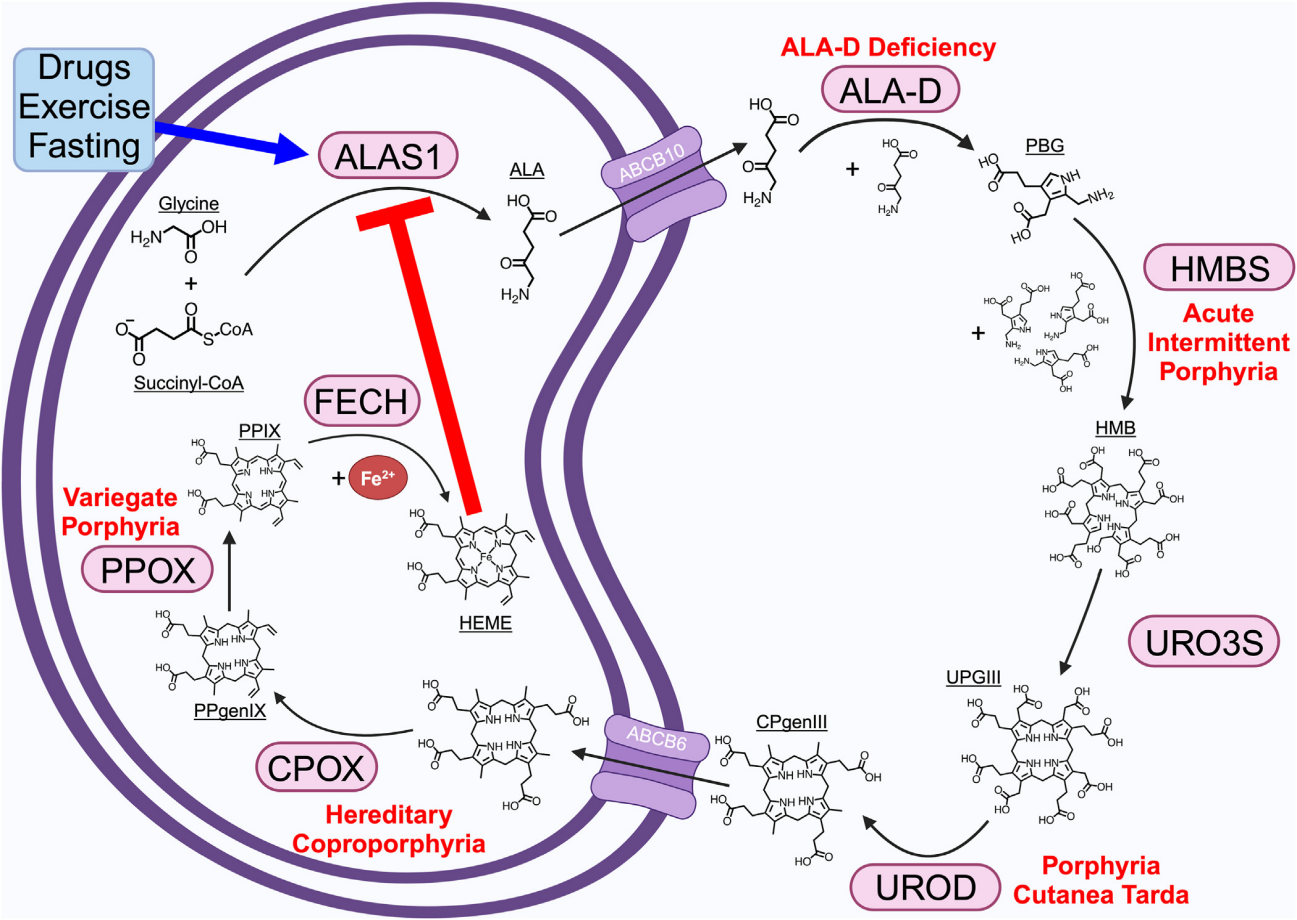
**Heme synthesis in nonerythroid cells.** Heme is generated in nonerythroid cells through a series of eight reactions facilitated by both mitochondrial (ALAS1, CPOX, PPOX, FECH) and cytosolic enzymes (ALA-D, HMBS, URO3S, UROD). This requires the transport of heme precursors to be transported across the mitochondrial membrane by ABCB10 and ABCB6. ALAS1 is the rate-limiting enzyme to this pathway and is therefore susceptible to the highest degree of regulatory feedback. ALAS1 experiences strong negative regulation by heme, which disrupts the enzyme’s expression from the transcriptional to the posttranslational level. Stimulators of heme protein expression (*i.e.*, drugs, exercise, fasting) induce expression of ALAS1. Deficiencies or defects in any of the heme biosynthetic enzymes, other than ALAS1, lead to conditions called porphyrias. Amongst the porphyrias, five of them (each highlighted in red next to the enzyme whose defect causes it) drive pathologies specifically in nonerythroid tissues. ALAS, *δ*-aminolevulinic acid synthase; ABCB, ABC subfamily B member; ALA, *δ*-aminolevulinic acid; ALA-D, ALA dehydratase; CPgenIII, coproporphyrinogen III; CPOX, coproporphyrinogen oxidase; FECH, ferrochelatase; HMB, hydroxymethylbilane; HMBS, HMB synthase; PBG, porphobilinogen; PPgenIX, protoporphyrinogen IX; PPIX, protoporphyrin IX; PPOX, protoporphyrinogen oxidase; UPGIII, uroporphyrinogen III; URO3S, uroporphyrinogen III synthase; UROD, uroporphyrinogen decarboxylase; .

The process of heme synthesis has been extensively reviewed and occurs in eight steps taking place between the mitochondria and cytosol (11, 12, 13, 14, 15). This process is summarized in Figure 1. Beginning within the mitochondria, the first and rate-limiting step of heme biosynthesis involves the condensation of succinyl-CoA and glycine into *δ*-aminolevulinic acid (ALA) *via* ALAS. Two isoforms of ALAS exist: ALAS1, which is a ubiquitously expressed enzyme, and ALAS2, which is specific to erythrocytes (16, 17). In the cytosol, two molecules of ALA are converted into a monopyrrole, porphobilinogen, *via* ALA dehydratase (ALA-D). Four molecules of porphobilinogen are then converted into a linear tetrapyrrole, hydroxymethylbilane (HMB), through HMB synthase (HMBS). While HMBS is encoded by the same gene in both erythroid and nonerythroid cells, the gene itself contains an erythroid-specific promoter as well as a ubiquitous promoter, allowing for differential gene regulation between erythroid and nonerythroid cells (18). Cyclization of HMB into uroporphyrinogen III (UPGIII) is catalyzed by uroporphyrinogen III synthase (URO3S), thereby preventing the spontaneous cyclization of HMB into unusable uroporphyrinogen I (19, 20). Uroporphyrinogen decarboxylase (UROD) then catalyzes the decarboxylation of uroporphyrinogen III to generate coproporphyrinogen III (CPgenIII), which is subsequently transported back into the mitochondria. In the mitochondria, CPgenIII is converted into protoporphyrinogen IX and then PPIX through the respective actions of coproporphyrinogen oxidase (CPOX) and protoporphyrinogen oxidase. Finally, ferrochelatase (FECH) inserts ferrous iron into PPIX to complete the heme biosynthetic process.

Many proteins outside of the core heme biosynthetic enzymes have been identified to be essential for heme biosynthesis. Among such proteins, many have functions related to iron homeostasis. Mitoferrin (MFRN) is a mitochondrial iron transporter, which exists in two paralogous forms: MFRN1 and MFRN2. While MFRN1 mediates iron transport into the mitochondrial matrix of erythrocytes to complete heme synthesis, MFRN2 performs this role ubiquitously, with its knockdown greatly reducing iron incorporation into heme *in vitro* (21, 22). Proteins associated with iron sulfur cluster (ISC) biogenesis are also essential for heme synthesis. The heme biosynthetic enzymes ALA-D and FECH are both ISC proteins, meaning that ISC synthesis and transport are required for both of their functions and therefore the procession of heme synthesis (23, 24). Until recently, ABC subfamily B member 7 (ABCB7), which functions as a mitochondrial ISC exporter, and glutaredoxin 5, which participates in ISC assembly, have only been shown to participate in erythroid heme synthesis (25, 26). However, interactions between FECH and both ABCB7 and glutaredoxin 5 has recently been reported in nonerythroid cells, supporting a potential role for them in nonerythroid heme synthesis (27).

Other key mediators of heme synthesis include certain porphyrin intermediate transporters, which function by transporting porphyrin intermediates between mitochondrial and cytosolic compartments. One such transporter is ABCB6. ABCB6 binds and transports porphyrins, with the transporter having the greatest affinity for coproporphyrin III, a stable derivative of CPgenIII (28, 29, 30). As CPgenIII requires transport into the mitochondria for heme synthesis to proceed and silencing of *Abcb6* inhibits heme synthesis in both erythroid and nonerythroid cells (28), it seems likely that ABCB6 functions to some extent as a CPgenIII transporter required for the procession of heme synthesis. Another transporter, ABCB10, is important in the early steps of nonerythroid heme biosynthesis. It has been shown that silencing of *Abcb10* reduces cellular porphyrin levels in rat cardiac cells, and that this effect can be rescued with ALA treatment. With ALAS1 protein levels unchanged, it was postulated that this transporter is required in heme synthesis for either ALA production or export out of the mitochondria (31). ABCB10 is increased in cell culture models, mouse models, and patient samples of ischemic cardiomyopathy resulting in increased heme concentrations (31). This upregulation is likely protective, as hearts from *Abcb10*^+/−^ fare worse after ischemia reperfusion injury (32). While it is not clear exactly why these mice fare worse, the authors speculate it may be due to impaired function of heme-dependent redox proteins such as catalases and peroxidases (31).

### Regulation of heme synthesis

Erythroid and nonerythroid heme synthesis utilize very similar proteins to facilitate an identical set of reactions. However, despite this likeness, the amount of synthesis between erythroid and nonerythroid cells varies greatly to account for the especially high heme requirement in erythrocytes (4). This rate difference can be attributed to different regulatory factors, many of which affect ALAS expression. It has been extensively shown that heme negatively regulates ALAS1 expression from the transcriptional to the posttranslational level (7, 17, 33, 34, 35, 36, 37, 38, 39). Heme functions as a stimulatory ligand for the nuclear receptors REV-ERBα and REV-ERBβ (40). REV-ERBα reduces the expression of PPARG coactivator 1α, a positive regulator of ALAS1 expression, thereby acting in a negative feedback loop to inhibit its own production (35, 40). Heme also mediates the regulation of ALAS1 by the circadian clock transcriptional complex, basic helix-loop-helix ARNT-like 1-neuronal PAS domain protein 2. When intracellular heme levels are low, the basic helix-loop-helix ARNT-like 1–neuronal PAS domain protein 2 transcriptional complex is active, upregulating the expression of *Alas1*. However, when heme levels rise, this induction is lost (7). Beyond the transcriptional level, heme has also been shown to reduce *Alas1* mRNA stability, prevent ALAS1 protein targeting to the mitochondria, and mediate the degradation of mitochondrial ALAS1 through the proteases, ClpXP and LONP1 (33, 36, 37, 38, 39).

Heme has also been shown to regulate the erythroid ALAS, with the ALAS2 precursor protein containing heme regulatory motifs that prevent its translocation into the mitochondria when bound to heme (37). However, erythropoietic regulators and iron availability have proven to be the primary regulators of ALAS2 (41, 42). This absence of a robust negative feedback loop between ALAS2 and heme likely accounts for heme synthesis levels being far greater in erythrocytes *versus* their nonerythroid counterparts.

Nonerythroid heme synthesis can also be regulated by stimuli that affect the expression of hemoproteins. For example, certain drugs that induce cytochromes P450 in the liver also induce the expression of *Alas1* (33, 43, 44). This induction is mediated through drug-responsive enhancers existing upstream of the *Alas1* gene, which have been shown to be activated upon exposure to drugs such as phenobarbital, glutethimide, metyrapone, and propylisopropylacetamide (43, 44, 45). Additionally, stimulators of mitochondrial biogenesis, such as exercise training and fasting, increase *Alas1* expression in skeletal muscle and hepatocytes, respectively (46, 47). The latter induction is mediated through PPARG coactivator 1α, NRF1, and FOXO1 (47).

ALAS1 expression and the resulting heme synthesis are crucial for metabolic function. Doxorubicin induces cardiomyopathy, in part, through the downregulation of ALAS1 driving mitochondrial iron overload and subsequent ferroptosis. It is worth noting that ALAS1 knockdown in cardiomyocytes *in vitro* was sufficient to increase mitochondrial iron but did not induce ferroptosis (48). *In vivo*, genetic deficiency of ALAS1 was shown to reduce mitochondrial function, impair glucose tolerance, and drive insulin resistance in aged mice (49). This age-specific phenotype may be explained by the reduction in ALAS1 enzymatic activity observed with aging (50). A complete loss of *Alas1* at the organismal level has proven to be embryonically lethal, suggesting the enzyme’s necessity in one or more essential biological processes, which likely includes mitochondrial biogenesis and function (10). Inhibition of heme synthesis also impairs embryonic stem cell fate indirectly as a result of succinate accumulation that occurs as a result of succinyl-CoA metabolism by ALAS1 or by reduced succinyl dehydrogenase abundance, a heme-dependent enzyme (51).

Aside from ALAS1, deficiency or defects in any of the other heme biosynthetic enzymes can lead to porphyrias, which are disorders related to disturbances in heme metabolism (Fig. 1). Porphyrias can be divided based on whether their effect is most largely felt in erythroid *versus* nonerythroid cells. Amongst the nonerythroid porphyrias are ALA-D deficiency, acute intermittent porphyria, porphyria cutanea tarda, hereditary coproporphyria, and variegate porphyria (52). These conditions are respectively caused by defects or deficiencies in ALA-D, HMBS, UROD, CPOX, and protoporphyrinogen oxidase (52). Aside from porphyria cutanea tarda, the nonerythroid porphyrias can be associated with acute porphyria attacks triggered by stressors that promote heme synthesis (*e.g.*, drugs, fasting) (52). These attacks arise from the accumulation of heme precursors in nonerythroid tissues and cause variable symptoms (*e.g.*, abdominal pain, nausea, vomiting, peripheral neuropathy, and/or seizures), depending on the enzyme deficiency (52). Because porphyrins are photoreactive, patients with porphyrias experience symptoms such as irritation, redness, or pain within minutes of sun exposure (4). Patients experiencing an acute attack are commonly treated with intravenous heme or glucose, both of which negatively regulate ALAS1 (53). Similar symptoms of acute porphyrias are observed with lead (Pb) poisoning due to inhibition of ALA-D and ALA accumulation (54). Lead displaces zinc from the metal binding sites of ALA-D making it a major target of lead poisoning but lead can also inhibit CPOX and FECH (55). Overall, the consequences associated with porphyrias highlight the importance of nonerythroid heme synthesis occurring to completion and acting to maintain heme levels such that cellular needs are met.

## Intracellular transport to hemoproteins

### Mitochondrial export

**Figure 2:**
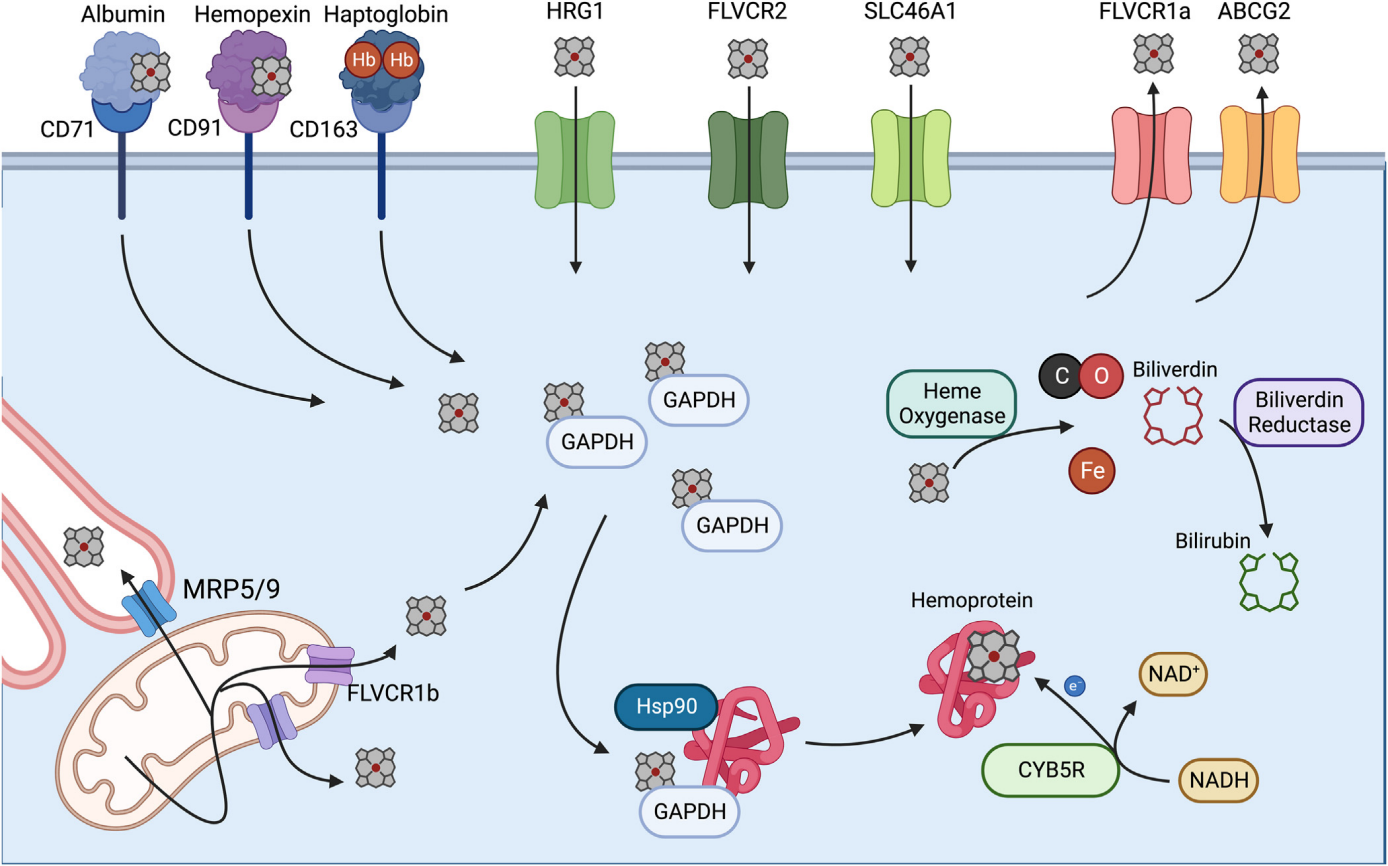
**Overview of heme metabolism.** Heme synthesis occurs in the mitochondria and is exported out of the mitochondria by FLVCR1b or PGRMC1. Heme enters the secretory system through MRP5/9 at points of contact between the endoplasmic reticulum and mitochondria. Heme that is exported into the cytosol is buffered and shuttled by GAPDH which facilitates insertion of heme into hemoproteins with chaperone proteins such as HSP90. In many cases, the redox state of the hemoprotein is maintained by CYB5R. Excess heme is catabolized by heme oxygenase, which produces iron, carbon monoxide, and biliverdin. Biliveridin is then further reduced to bilirubin by biliverdin reductase. Several mechanisms of heme import have been proposed including the endocytosis of heme in complex with serum albumin by CD71, in complex with hemopexin by CD91, as hemoglobin in complex with haptoglobin by CD163, and imported as free heme by HRG, FLVCR2, or HCP1/PCFT. FLVCR1a and ABCG2 have been proposed to mediate heme export. ABCG2, ABC subfamily G member 2; CYB5R, cytochrome b5 reductase; FLVCR, feline leukemia virus subgroup C receptor; HRG, heme responsive gene 1; HSP90, heat shock protein 90; MRP 5/9, multidrug resistance proteins 5/9; PCFT, protein-coupled folate transporter; PGRMC1, progesterone receptor membrane component 1; SLC46A1, solute carrier family 46 member 1.

Once synthetized, heme must traverse both the inner and outer mitochondrial membranes to reach nonmitochondrial hemoproteins. The involvement of the “b” isoform of the feline leukemia virus subgroup C receptor 1 (FLVCR1) gene (FLVCR1b) in this process has been suggested (56). The FLVCR family of proteins has been proposed to act as heme importers and exporters. The FLVCR1 isoforms, FLVCR1a and FLVCR1b, export heme across the plasma membrane and mitochondrial membrane, respectively (56, 57, 58, 59), and FLVCR2 has been proposed as a heme importer localized to the plasma membrane (60). FLVCR1b has been shown to mediate mitochondrial heme export in both erythroid and nonerythroid cells (Fig. 2) (56, 57). Unfortunately, FLVCR1b is only distinguished from FLVCR1a by an alternative transcriptional start site, thereby limiting the use of genetic tools to target FLVCR1b independent of FLVCR1a making it difficult to study this isoform *in vivo* (61).

Progesterone receptor membrane component 1 (PGRMC1) has also been proposed to facilitate the transport of heme from the mitochondria to hemoproteins. Several attributes make PGRMC1 a good candidate for this role. PGRMC1 has been shown to interact with FECH in both erythroid and nonerythroid cells and localize to the outer mitochondrial membrane, suggesting it spans mitochondrial membranes (27, 62). Additionally, PGRMC1 is able to transfer its heme to apo-proteins in isolated protein preparations (62). Finally, PGRMC1 has been found at contact points between the mitochondria and endoplasmic reticulum (ER), which would allow it to facilitate heme transfer from the mitochondria to the secretory system (63). However, uncertainty remains as it is unclear how PGRMC1 may facilitate heme transfer across both membranes. Additionally, some of PGRMC1’s functions such as stabilizing cytochrome proteins are independent of PGRCM1’s ability to bind heme (64). Further work is necessary to fully understand the role of PGRMC1 in heme biology.

### Transport into the secretory pathway

Most hemoproteins are synthesized, processed, and folded within the ER and Golgi complex, where they subsequently enter the secretory pathway to reach their designated destinations, such as the plasma membrane, lysosomes, or peroxisomes (65). Hence, there must be specific systems mediating heme transfer among these distinct compartments. Interorganelle membrane contact sites have been demonstrated to play a role in heme transfer throughout the secretory pathway (66). Studies in *Saccharomyces cerevisiae* using fluorescent heme sensors revealed heme flux between mitochondria and the nucleus is regulated by mitochondrial dynamics and ER-mitochondria contact sites (67). Multidrug resistance proteins 5 (MRP5/ABC subfamily C member 5) is a member of the ABCC subfamily of transporters (68) that is expressed on both the plasma membrane and endosomal compartments and has been proposed to regulate heme levels in the mammalian secretory pathway (Fig. 2) (69). MRP5 was first described as a heme transporter in *Caenorhabditis elegans* where it exports heme from the intestine to extraintestinal tissues. Further studies confirmed MRP5’s role in regulating systemic heme homeostasis in zebrafish embryos and embryonic fibroblasts isolated from MRP5 null mice (69). The paralog MRP9 has been proposed to have a similar function, thus compensating for MRP5’s absence. Double knock out of MRP5 and MRP9 results in an impaired reproductive phenotype in male mice due to impaired heme handling and mitochondrial dysfunction in sperm (70). The lack of further viability and reproductive phenotypes in these mice suggests the presence of other redundant pathways. More detailed analysis of the somatic cells in these KO mice is warranted to better understand the importance of these proteins in heme trafficking *in vivo*.

### Insertion into hemoproteins

Recently, several groups have investigated the role of GAPDH as a heme buffer and shuttle in the cytosol. GAPDH binds heme with a conserved histidine (H53 in human GAPDH) (71). Deletion of TDH3, which encodes an isoform of GAPDH in yeast, or mutation of the conserved histidine increases cytosolic labile heme, thus demonstrating the enzyme’s buffering capacity (71, 72). Despite this increase in available heme, the heme-dependent transcription factor Hap1 has reduced activity suggesting impaired heme insertion (71, 72). GAPDH therefore likely acts to both buffer and shuttle heme to apo-hemoproteins. Similar studies have shown the importance of GAPDH in heme insertion into soluble guanylate cyclase beta (sGCβ), inducible nitric oxide synthase (NOS), neuronal NOS, tryptophan 2-3-dioxygenase (TDO), indoleamine 2,3-dioxygenase (IDO), and globins (73, 74, 75, 76, 77, 78). In most of these cases, heme insertion into the apoprotein is facilitated by chaperone proteins such as heat shock protein 90 and alpha hemoglobin stabilizing protein (Fig. 2) (74, 75, 79, 80, 81).

It was previously thought that hemoproteins exist predominantly in the holo-enzyme form, but more recently it has been appreciated that there is a large pool of hemoproteins which exist in their apo form (79). This view presents heme insertion as an important posttranslational modification, allowing cells to rapidly increase the activity of hemoproteins without increasing gene expression. IDO and TDO are perhaps the best-characterized examples of this mechanism (5, 6). For both enzymes, their substrate, tryptophan, increases their affinity for heme and subsequently their enzymatic activity. A similar model has now been proposed for sGC. sGC is the primary receptor for nitric oxide (NO), which binds to the heme in the beta subunit. NO promotes heme incorporation into sGCβ, thereby promoting its association with sGCβ and downstream signaling (82). This, however, may be more than an example of a ligand promoting hemoprotein maturation. Rather, it may hint at a more general mechanism by which NO regulates labile heme and heme allocation. NO increases labile heme (72) and has a bimodal effect on heme insertion (74, 83). Evidence suggests this is due to the formation of heme-NO (84), but more work is needed to fully understand this phenomenon.

Thus far, work in this field has been primarily performed *in vitro*. Cell culture models and purified enzyme preparations provide controlled environments with minimal variables to investigate hemoprotein assembly, but these tools are limiting when considering implications for (patho)physiology. It remains to be fully appreciated how these pathways play a part in whole animal physiology or disease states. Given the importance of NO in a broad range of physiologic systems (*e.g.*, cardiovascular physiology (85), host immune response (86), cerebral health (87), and water and electrolyte homeostasis (88)), it is tempting to hypothesize a variety of physiologic implications for these pathways. Heme-NO has recently been reappreciated as a direct activator of apo-sGC (89, 90, 91), but the studies outlined here suggest this phenomenon is just a small part of NO regulating hemoproteins more broadly. It has been hypothesized NO regulation of TDO and IDO heme insertion is a potential mechanism by which tryptophan metabolism is regulated in inflammation, asthma, and cancer biology (74). Heme trafficking and insertion into hemoproteins is a much more dynamic process than is widely appreciated and is worth consideration and investigation in cell signaling and physiology more broadly.

## Regulation of redox state

The heme oxidation state is critical for hemoprotein maturation and function. In many cases, the cycling of this oxidation state is intrinsic to the function of the hemoprotein. For example, in the electron transport chain, cytochrome *c* is reduced to Fe^2+^ by complex III and oxidized to Fe^3+^ by complex IV as it facilitates electron transfer between these complexes (92). For other hemoproteins, the cycling of the redox state is controlled by the enzyme itself. For example, NOS must be in its ferrous state to bind its substrate, oxygen. During the enzymatic cycle, the heme becomes oxidized twice and must be reduced by electrons from the electron donors NADPH and BH_4_ (93). The final reduction is necessary to reset the heme to its ferrous form allowing the cycle to begin again. In this example, heme reduction is facilitated by the reductase domain of NOS itself rather than a partner reductase. Heme redox state can also dictate the binding of heme to the hemoprotein. This is the case for REV-ERBβ, which preferentially binds Fe^3+^ heme despite the role of REV-ERBβ as a gas sensor (8). Rather than being reduced by reductase enzymes or domains as in the above cases, the heme undergoes electrochemical reduction in the presence of these gases, allowing CO or NO to bind (8).

Because the redox state is important for heme function, aberrant heme oxidation can disrupt hemoprotein function. Perhaps the most well-known and described example is that of hemoglobin. In physiologic conditions, hemoglobin exists in the ferrous state and binds oxygen. However, under oxidative or nitrosative stress hemoglobin can become oxidized to ferric methemoglobin (94). Methemoglobin does not bind oxygen and must be reduced back to its ferrous state by cytochrome b5 reductase 3 (CYB5R3) to prevent methemoglobinemia, a life-threatening condition (95).

The CYB5R family of proteins reduces a host of hemoproteins and protects against oxidative stress in both erythroid and nonerythroid cells (96). The importance of these proteins in regulating specific targets is perhaps best exemplified in the regulation of vascular NO signaling by CYB5R3. In resistance arteries, endothelial cells express the alpha chain of hemoglobin, which scavenges NO under oxygenated conditions (97, 98, 99, 100). CYB5R3 is necessary for maintaining alpha chain of hemoglobin in the Fe^2+^ state allowing for efficient scavenging of NO (97). In the smooth muscle, CYB5R3 regulates NO signaling through maintaining sGC, the receptor for NO, in a reduced state (101). This has been shown to be particularly important in diseases with oxidative stress, such as hypertension and sickle cell disease (102, 103). Studies using KO mice have been useful in elucidating the physiological importance of this family of enzymes in a variety of tissues. CYB5R is critical for pancreatic insulin secretion (104), heart function (105), liver lipid metabolism (106), cerebral function (107), and vascular control of blood pressure (102). While these studies have been helpful for investigating the functional importance, few of these have investigated the specific targets of CYB5R in these systems. Identifying these targets will provide exciting insight to the role of hemoproteins in each of these tissues.

## Intercellular trafficking

### Import

Heme trafficking within and between cells is a complex process involving many molecular players and transporters. While each cell has the capacity to independently produce the heme it needs through *de novo* synthesis, heme can also be acquired from extracellular sources through two distinct mechanisms: receptor-mediated endocytosis of heme– or hemoglobin–protein complexes and the uptake of free heme through specific plasma membrane transporters (59). The first mechanism involves the plasma proteins serum albumin, hemopexin (HPX), or haptoglobin. Serum albumin has at least two binding sites for heme and was considered to function as a buffering system, enabling the transfer of heme to HPX (59). HPX, with its highest affinity, then facilitates the uptake of heme by the LDL receptor–related protein 1 (CD91) (108, 109). Recent findings indicate that the heme–albumin complex can be directly taken up by cells through the transferrin receptor 1 (CD71) *in vitro* (109). However, the physiological significance of this system *in vivo* is yet to be fully understood. Additionally, heme in the form of hemoglobin binds haptoglobin and is taken up by CD163 (Fig. 2) (110). These systems play a critical role in scenarios characterized by increased intravascular hemolysis by ensuring the proper uptake and breakdown of heme/hemoglobin. This, in turn, helps prevent heme-induced tissue damage, as confirmed by studies in KO mouse models. For instance, mice lacking HPX exhibit severe endothelial dysfunction and oxidative damage in cardiac and hepatic tissues when exposed to excessive heme (111, 112). Similarly, supplementing HPX in murine models of sickle cell disease has been shown to ameliorate cardiovascular damage (113), inhibit proinflammatory polarization of macrophages, and preserve the phagocytic capacity of macrophages (112, 114, 115).

Conversely, there is ongoing debate regarding the capability of cells to take up free heme, which refers to heme not bound to proteins. Initially, the first suggested heme transporter was solute carrier family 46 member 1 (also known as heme carrier protein 1 and protein-coupled folate transporter), which is expressed in duodenal enterocytes and was originally believed to play a role in absorbing dietary heme (Fig. 2) (116). However, subsequent research revealed that solute carrier family 46 member 1 exhibits a stronger affinity for folate than for heme and functions as a proton-coupled, high-affinity folate transporter (117). Furthermore, mutations in this gene were identified in individuals with hereditary folate malabsorption syndrome (117, 118). It remains to be determined whether this transporter can effectively transport both folate and heme based on the body's requirements.

As mentioned above, FLVCR2 has also been proposed as a heme importer (60). Studies have demonstrated that FLVCR2 plays a specific role in endothelial cells during the process of sprouting angiogenesis (119). Consequently, the deletion of FLVCR2 in mice leads to lethality in late gestation, primarily due to the improper formation of blood vessels within the central nervous system (120). However, it is worth noting that the potential impact of altered heme metabolism on these observed phenotypes has not been thoroughly investigated. Furthermore, a recent study reported that FLVCR2 can be found within mitochondria, where it functions as a heme sensor to regulate thermogenesis (121).

Finally, studies conducted in the heme auxotroph nematode *C. elegans* have resulted in the discovery of heme responsive genes (HRGs), a group of transporters involved in heme trafficking across cellular membranes (65). A compelling candidate for intestinal heme absorption is HRG1, a four-transmembrane-domain heme transporter expressed in the human small intestine, where it could function as a heme importer *via* endocytic compartments (122). HRG1 is also highly expressed on erythrophagosomal membranes, where it imports heme into the cytosol of macrophages during iron recycling from erythrocytes (123, 124).

### Export

Export of heme can be mediated by plasma membrane exporters. FLVCR1a, which is a transmembrane protein belonging to the major facilitator superfamily of transporters, controls the intracellular the heme pool by modulating heme efflux from the cytosol toward the extracellular environment (Fig. 2) (58). Moreover, FLVCR1a is needed to sustain proper heme synthesis, being part of a coordinated functional axis together with the heme synthesis rate-limiting enzyme ALAS1 (125). By coordinating the amount of heme required during globin synthesis, the role of FLVCR1a is essential in erythroid precursors (57, 126, 127). However, numerous studies have highlighted its relevance in maintaining the homeostasis of various other tissues, including the intestine (128), liver (129), vascular system (130, 131), and peripheral nervous system (132, 133, 134, 135). Collectively, mounting evidence indicates that the regulation of heme metabolism mediated by FLVCR1a plays a crucial role in meeting the energy needs of highly proliferative cells. In line with this perspective, FLVCR1a exhibits high expression levels during embryonic development (131, 133, 134, 135, 136) and in cancer cells (137, 138).

Another protein with a proposed role in heme efflux is ABC subfamily G member 2 (ABCG2). This is a member of the ABC transporter family with a proven ability to bind heme (139, 140). Studies in mice show that ABCG2 abrogation leads to PPIX accumulation in erythroid cells (141). ABCG2 is also expressed in other cell types, including hepatic canalicular membranes, renal proximal tubules, intestinal epithelium, and placenta (142). Moreover, ABCG2 expression is induced in HeLa cells after the stimulation of heme synthesis (56). These results might suggest a general role for ABCG2 in the heme export process, even though the substrate specificity under physiological or pathological conditions is still unclear.

Interestingly, some transporters with heme-related functions have been studied in cellular crosstalk and metabolic cooperation among cells (59, 138). For instance, FLVCR2 has been proposed as a prognostic biomarker for acute myeloid leukemia due to its implication in the modulation of the tumor microenvironment in acute myeloid leukemia patients, specifically in terms of immune infiltration degree and cancer cell proliferation rate (143). On the other hand, FLVCR1a function in tumor-associated endothelial cells has been shown to affect the composition of the tumor microenvironment, having an impact on the metabolic setting of cancer cells (144).

These studies suggest the intriguing possibility that intercellular heme trafficking, extending beyond heme movement within cells, may serve important biological functions. What remains to be clarified is the extent to which these observed phenotypes are linked to the heme-transporting role of the proteins, or conversely, if they stem from additional and yet unknown functions of these transporters. Notably, recent findings have revealed that both FLVCR1a and FLVCR2 also function as high-affinity choline importers (145, 146). Supplementation of choline was effective in rescuing the proliferation defect seen in FLVCR1 KO cells grown in choline-deficient conditions and partially mitigating the lethal embryonic phenotype observed in Flvcr1 null mice (145). These discoveries raise the intriguing question of potential interactions between heme and choline metabolism. Subsequent investigations are necessary to determine whether the alterations in heme metabolism associated with FLVCR1 and FLVCR2 are a direct result of their roles as heme transporters or are indirectly influenced by changes in choline metabolism.

## Heme degradation

Heme is a hydrophobic and highly reactive molecule. Free heme catalyzes the formation of hydroxyl radicals, resulting in lipid oxidation, protein damage, and promotes cell death. Additionally, its hydrophobicity allows it to intercalate into cellular membranes further, promoting membrane lipid oxidation (59). As such, heme degradation is a crucial component of heme metabolism that has been studied extensively over many decades (147, 148, 149, 150, 151, 152). Over the course of three reactions, heme oxygenase (HO) catalyzes the breakdown of heme into biliverdin, carbon monoxide (CO), and free iron (Fig. 2). Such reactions have been described in great detail (13, 150, 151). Within the first, HO forms a complex with ferric heme, which undergoes reduction by NADPH-cytochrome P450 reductase to become ferrous heme. Molecular oxygen then binds to ferrous heme iron and undergoes a subsequent reduction and protonation (process hereafter referred to as oxygen activation). Oxygen activation drives the formation of a reactive intermediate (Fe^3+^-OOH), which facilitates the cleavage of the α-*meso*-carbon of the heme porphyrin ring to form hydroxyheme. In the second reaction, oxygen binding and activation occurs again to facilitate the conversion of ferric hydroxyheme to ferrous verdoheme and CO. Ferrous verdoheme then undergoes one final round of oxygen binding and activation, with the reactive intermediate formed driving the cleavage of the heme porphyrin ring to generate ferric iron biliverdin. The release of both free ferrous iron and biliverdin occurs after ferric biliverdin is reduced once again by NADPH-cytochrome P450 reductase. Biliverdin is then metabolized to bilirubin by biliverdin reductase (153). CO, biliverdin, and bilirubin have all been reported to act as signaling molecules in their own right and exert protective effects (154, 155). This set of reactions is assumed to occur ubiquitously in respirating organisms as all cell types express, and therefore require the regulation of heme.

Two isoforms of HO have been implicated in heme catabolism: HO-1 and HO-2 (149, 156). These isozymes share around 43% similarity in primary structure and are mechanistically identical in how they degrade heme (149, 156). However, they do tend to vary in regard to substrate affinity and reaction rates, with HO-1 showing higher affinity for heme and an increased V_max_ relative to HO-2 (149). This variation in catalytic efficiency makes sense given the contextual differences in which these enzymes are expressed. HO-1 is an inducible enzyme that is expressed in response to many cellular stressors, including heme, endotoxins (*i.e.*, lipopolysaccharide [LPS]), NO, UV radiation, and hypoxia (157, 158, 159, 160, 161, 162). Because of this, much of the existing literature describing HO-1 function has illustrated the enzyme’s major role in responding to oxidative stress and pathological states, such as in ischemia-reperfusion injury, atherosclerosis, and metabolic disease (157, 158, 159, 163, 164, 165). HO-1 has also been shown to enhance tumor growth and metastasis (166, 167). However, having elevated rates of heme degradation also increases the tumor’s dependence on heme synthesis to maintain adequate heme levels (166). Mediating the same reactions as HO-1, HO-2 has been shown to be protective in response to hyperoxia, tissue injury, metabolic disease, and oxidative stress in the brain (168, 169, 170, 171). However, unlike HO-1, HO-2 is constitutively expressed and resistant to all stimuli except for glucocorticoids (172). Because of this variation in expression patterns, it seems likely that HO-2 mediates heme degradation under basal conditions, while HO-1 facilitates the majority of heme degradation during times of cellular stress.

Despite catalyzing the same reaction, HO-1 and HO-2 have several attributes which set them apart from each other (Table 1). Encoded by *Hmox1*, HO-1 can be induced throughout all tissue types but is most highly expressed within the spleen and liver (173). These tissues are similar in their enrichment for phagocytes that facilitate erythroid cell recycling, a process which makes the largest contribution to total heme degradation within an organism (13, 174). The requirement for HO-1 in this process is highlighted by the fact that global *Hmox1*^*−/−*^ mice are depleted of erythroid-recycling phagocytes, leading to elevated bouts of erythrocyte death and subsequent damage to the surrounding vasculature (175). At the subcellular level, HO-1 has been shown to localize to the ER, mitochondria, caveolae, and nucleus (147, 176, 177, 178). The enzyme is thought to catabolize heme in each of these locations, except for the nucleus, where HO-1 activity is lost as a result of it being truncated for transport into the nucleus (178). The role of nuclear HO-1 is not entirely clear, but it may modulate the activity of transcription factors by direct interactions (178).

**Table 1:** Comparison of heme oxygenase 1 and 2

Attribute	HO-1	HO-2
Expression	Inducible	Constitutive
Tissue enrichment	Spleen, liver	Testis, brain
Subcellular localization	ER, mitochondria, caveolae, nucleus	ER, cytoplasm
Catalytic efficiency	Greater	Lesser
Induced by	Heme, endotoxins, nitric oxide, UV radiation, hypoxia, + more	Glucocorticoids
Function	Degrade heme in response to cellular stress	Act as a buffer for labile heme; degrade heme under basal conditions?
Regulation by heme	Derepresses BACH1 to drive induction	Prevents enzyme targeting for degradation

BACH, BTB domain and CNC homolog; endoplasmic reticulum.

Outside of HO-1’s role in responding to cellular stresses, the enzyme’s more obvious function is in regulating intracellular heme levels. *In vivo* induction of HO-1 reduces mitochondrial heme content in the liver of rats, and this effect is blocked by an HO-1 inhibitor, tin PPIX (176). An important regulatory step for controlling HO-1 induction and intracellular heme levels comes from the act of heme sensing. In the absence of a stimulus, the transcriptional repressor BTB domain and CNC homolog 1 (BACH1) will bind to an upstream enhancer for *Hmox1*, thereby preventing *Hmox1* expression (179). However, when heme levels rise, heme binds BACH1 and drives it’s unbinding from the enhancer region (179, 180, 181) This leaves the DNA accessible such that nuclear response factor 2 (NRF2), a transcription factor that promotes the expression of *Hmox1*, is able to bind (181, 182). In this negative feedback loop, heme drives the expression of its degrading enzyme to prevent its own excess intracellularly. There is some evidence cells take advantage of this regulatory mechanism to modulate HO-1, BACH1, and NRF2, thereby harnessing their antioxidant properties. For example, in murine bone marrow–derived macrophages, LPS stimulation increases labile heme concentrations, reduces BACH1 protein, and increases the expression of both NRF2 and HO-1 protein (183). This cycle also works in the other direction in a mouse model of lung cancer where the stabilization of NRF2 increases HO-1, thereby decreasing heme, increasing BACH1 protein, and promoting tumor metastasis (167).

Encoded by *Hmox2*, HO-2 is constitutively and ubiquitously expressed, with specific enrichment in the testes and brain (173). Subcellularly, HO-2 is mostly localized in the ER, though cytoplasmic expression of the enzyme has also been observed to a lesser extent (176). Structurally, HO-2 contains three different heme binding regions in its primary sequence, with one being within the enzyme’s active site and the other two being regulatory motifs (184, 185). While heme can bind the enzyme at all three locations, it appears that only active site binding can affect the catalytic function of the enzyme (185). Part of this may be due to the fact that heme binding in the active site prevents posttranslational breakdown of HO-2, which would play part in its capacity to catalyze the degradation of heme (186). The other two heme binding sites have been shown to act in the transfer of heme to and from the active site, with the directionality of this transfer likely being dependent on the cell’s requirement for heme (152). Mutations at these sites do not substantially impair enzyme activity, suggesting that the role of these sites in heme degradation is not vital (185).

To date, HO-2’s role in heme degradation is described as only being required when free heme is present at levels beneath the threshold of HO-1 induction (187). However, this requirement has not yet been proven due to endogenous heme concentrations being too low under basal conditions to detect changes after modulating HO-2 expression or activity. Recently, a new function for HO-2 as a buffer for labile heme levels was identified in human embryonic kidney cells (187). This work showed that in modulating HO-2 levels, labile heme concentrations were altered in the opposite direction. HO-1 expression, HO activity, and total heme were all unaffected, suggesting that HO-2 is functioning to bind and buffer labile heme levels intracellularly (187). Altogether, the response to increasing heme levels begins with HO-2 until levels rise high enough to induce HO-1, which in turn, recovers heme back to homeostatic levels.

## Conclusions

Heme is often thought of as a stagnant prosthetic group of heme-dependent enzymes and proteins or is studied simply as a component of erythropoiesis. However, it is becoming increasingly clear that heme participates in nonerythroid cellular functions in a much more dynamic way. Functional studies into the pathways by which heme is metabolized and transported have revealed several examples (*e.g.*, heme participating in circadian rhythms (7), responding to NO signaling (72, 82), mediating the macrophage response to LPS (183), and increasing enzyme activity in response to substrate availability (5, 6)). This dynamic view of heme biology opens up exciting possibilities as to how heme may participate in a range of physiologic processes in nonerythroid cells.

## Conflict of interest

The authors declare that they have no conflicts of interest with the contents of this article.
